# How Do Virtual Visits Compare? Parent Satisfaction With Pediatric Diabetes Telehealth During the COVID-19 Pandemic

**DOI:** 10.3389/fcdhc.2021.794493

**Published:** 2022-01-05

**Authors:** Christine A. March, Radhika Muzumdar, Ingrid Libman

**Affiliations:** Division of Pediatric Endocrinology, University of Pittsburgh Medical Center (UPMC) Children’s Hospital of Pittsburgh, Pittsburgh, PA, United States

**Keywords:** type 1 diabetes, telehealth, telemedicine, COVID-19 pandemic, health care satisfaction

## Abstract

**Background:**

In response to the COVID-19 pandemic, many countries relaxed restrictions on telemedicine, allowing for a robust transition to virtual visits for routine care. In response, centers rapidly instituted and scaled telemedicine for pediatric diabetes care. Despite numerous center reports on their experience, little is known about parent perspectives on the widespread increase of telemedicine for pediatric diabetes appointments.

**Objective:**

To assess parent satisfaction with virtual care for pediatric diabetes during the COVID-19 pandemic.

**Methods:**

We conducted an online, cross-sectional survey of parents of youth with diabetes who receive care at a large, academic diabetes center regarding their perspectives on newly introduced virtual appointments. Parents were surveyed at two time points during the pandemic using a validated scale which was adapted for diabetes. We explored demographic and clinical factors which may influence parental satisfaction.

**Results:**

Overall, parents expressed high levels of satisfaction (>90%) with functional aspects of the visit, though only approximately half (56%) felt the visit was as good as an in-person encounter. Nearly three-quarters (74%) would consider using telemedicine again in the future. Prior use of telemedicine significantly influenced parent satisfaction, suggesting that parent preferences may play a role in continued use of telemedicine in the future. There was no difference in responses across the two timepoints, suggesting high satisfaction early in the pandemic which persisted.

**Conclusions:**

If permissive policies for telemedicine continue, diabetes centers could adopt hybrid in-person and virtual care models, while considering various stakeholder perspectives (providers and patients) and equity in access to virtual care.

## Introduction

Diabetes care lends itself well to telemedicine. Multidisciplinary providers can engage in virtual counseling for diabetes and nutrition education, and devices (e.g. glucometers, continuous glucose monitors, insulin pumps) can be shared with medical providers electronically *via* secure cloud systems. Indeed, telemedicine has led to short-term improvements in glycemic control in select populations (1) and enhanced youth engagement in their diabetes self-management (2). However, prior to the COVID-19 pandemic, use of telemedicine for routine diabetes care at pediatric diabetes centers was limited (3), with few published examples in the literature (1, 4, 5).

The COVID-19 pandemic led to a paradigm shift in routine care delivery with the widespread transition to virtual care when able. This was largely facilitated by the Center for Medicare and Medicaid Services (CMS) issuing telehealth waivers in March 2020. These waivers relaxed restrictive policies and allowed for virtual ambulatory care to be delivered direct to patients’ homes, resulting in an exponential increase in telemedicine encounters (6). Our large, academic diabetes center and others rapidly instituted and scaled virtual diabetes appointments beginning in March 2020 (3, 7). The objective of this study was to assess parent satisfaction with virtual pediatric diabetes care using a newly instituted diabetes tele-visit system.

## Methods

A cross-sectional online survey of parents of youth with type 1 diabetes was conducted to explore parent satisfaction with newly implemented telemedicine services during the COVID-19 pandemic. Detailed processes for the telemedicine visits were published elsewhere (7); in brief, nearly all aspects of diabetes appointments were transitioned to a virtual model. Patients continued to meet virtually with a diabetes provider to discuss various aspects of diabetes management and review blood glucose and insulin dosing records. To the best of our ability, ancillary care services essential to multidisciplinary care, including nutrition, diabetes education, and social work, were also maintained through virtual platforms. Families were asked to measure weight in advance of appointments and visit a local laboratory if bloodwork was needed for a hemoglobin A1c or routine screening labs. This study was deemed exempt by the University of Pittsburgh Institutional Review Board (PRO20070009).

### Survey

The 12-item Parent Satisfaction Survey, originally designed and validated to assess parent satisfaction with tele-mental health services, was used (8). Nine questions were retained, replacing the word “specialist” with “diabetes provider”. Three questions were eliminated (Telemedicine allowed my child to see a specialist sooner; My child would not have received services of a specialist without telemedicine; My child will receive the help he/she needs because of our telemedicine visit with the specialist) as they were felt to pertain to an initial consultation, and not the introduction of telemedicine into ongoing diabetes care. All items were scored on a 5-point Likert scale from strongly agree to strongly disagree.

An additional question was added to the survey asking parents about their perceived importance of different aspects of a diabetes visit to their child’s care, including checking growth, checking injection/pump sites, examining the child, reviewing device downloads in person, getting laboratory tests, and getting urine tests. Introductory questions also asked about the use of telemedicine before and during COVID-19, devices used to complete the visit (e.g. smart phone, computer), and internet access. Background characteristics were obtained, including child and family demographic information, diabetes diagnosis, management regimen, and most recent hemoglobin A1c. These questions were multiple choice with the option to include a short phrase clarifying any response choice of “other”.

Two parent stakeholders whose children have type 1 diabetes reviewed the survey for relevance and clarity to establish logical (face) validity. The shortened version of the Parent Satisfaction Survey, now applicable to diabetes care, was retested for internal reliability with a Cronbach’s alpha of 0.90, indicating a high degree of internal consistency without redundancy (9). The survey is provided in **Supplement 1**.

### Sample and Setting

Parents of children with type 1 or 2 diabetes from a large, academic diabetes center (UPMC Children’s Hospital of Pittsburgh), which provides care for over 2000 children with diabetes annually, were sent the survey. Eligible parents must have been able to answer the survey in English. A link to the web-based survey was distributed through the institution’s electronic health record (EHR) patient portal at two time points: first between August-September 2020 (following 1-2 telemedicine visits after the initial introduction of telemedicine services), and second in January-February 2021 (several months into telemedicine services). Survey data were collected using the University of Pittsburgh Qualtrics system.

### Analysis

Descriptive statistics were used to summarize participant characteristics and question responses. Sub-group analyses were defined *a priori* and included assessing differences by age (child less than 12 years vs adolescent 12 years and older), prior use of telemedicine services (yes/no), reported hemoglobin A1c at target (less than or equal to 7% vs greater than 7%), insulin pump use (yes/no), and primary insurance status (private/public) as a surrogate marker of socioeconomic status. Differences between groups were compared using Chi-Square, Fisher Exact, or Mann Whitney U Tests. All analyses were completed using STATA v15. For questions where parents were able to add a free text response (e.g. when selecting “other”), the short phrases were aggregated and summarized by author consensus.

## Results

Of 211 returned surveys, 115 (55%) were from August-September 2020 and 96 (45%) from Dec-Jan 2021. We excluded 43 surveys which were incomplete (n=40) or duplicates (n=3); an additional 9 were excluded as the child had not had a telemedicine visit, leaving 159 for analysis. Respondent characteristics are included in **Table 1**; these were similar to our clinic population for race, sex, and age, though more used continuous glucose monitoring and fewer had public insurance alone. The majority of respondents were mothers (n=140, 88%) from four different states. Only 18 (11%) had previously used telemedicine. Most respondents used a computer (n=102, 64%) or smart phone (n=41, 26%) for the visit, with few using a tablet (n=13, 8%). Only 3 respondents (2%) had no video capability or internet access and used a standard telephone for the visit.

**Table 1:** Child and household characteristics of survey respondents.

Characteristic	N = 159
*Child Characteristics*	
Age, years	13 [9-16]
Non-Hispanic white race	143 (90)
Male sex	87 (55)
Duration of diabetes, years	
<2	40 (25)
≥2	119 (75)
Recent hemoglobin A1c, %	
≤7	41 (26)
7.1 to <8	59 (37)
8.1 to <9	38 (24)
≥9	12 (8)
Not sure	8 (5)
Insulin pump use	102 (64)
Continuous glucose monitor use	149 (94)
*Household Characteristics*	
Household size	4 [4-5]
Parent education	
High school diploma	10 (6)
Some college	20 (13)
College degree	58 (36)
Graduate degree	68 (43)
Declined to answer	3 (2)
Household income (annual)	
<$50,000	24 (15)
$50,000 - <$100,000	48 (30)
≥$100,000	66 (42)
Declined to answer	21 (13)
Insurance status	
Private	18 (11)
Combined private/public	104 (66)
Public	30 (19)
Declined to answer	7 (5)
Qualifies for free lunch	35 (22)

Data are presented as N (%) or median [IQR].

**Figure 1:**
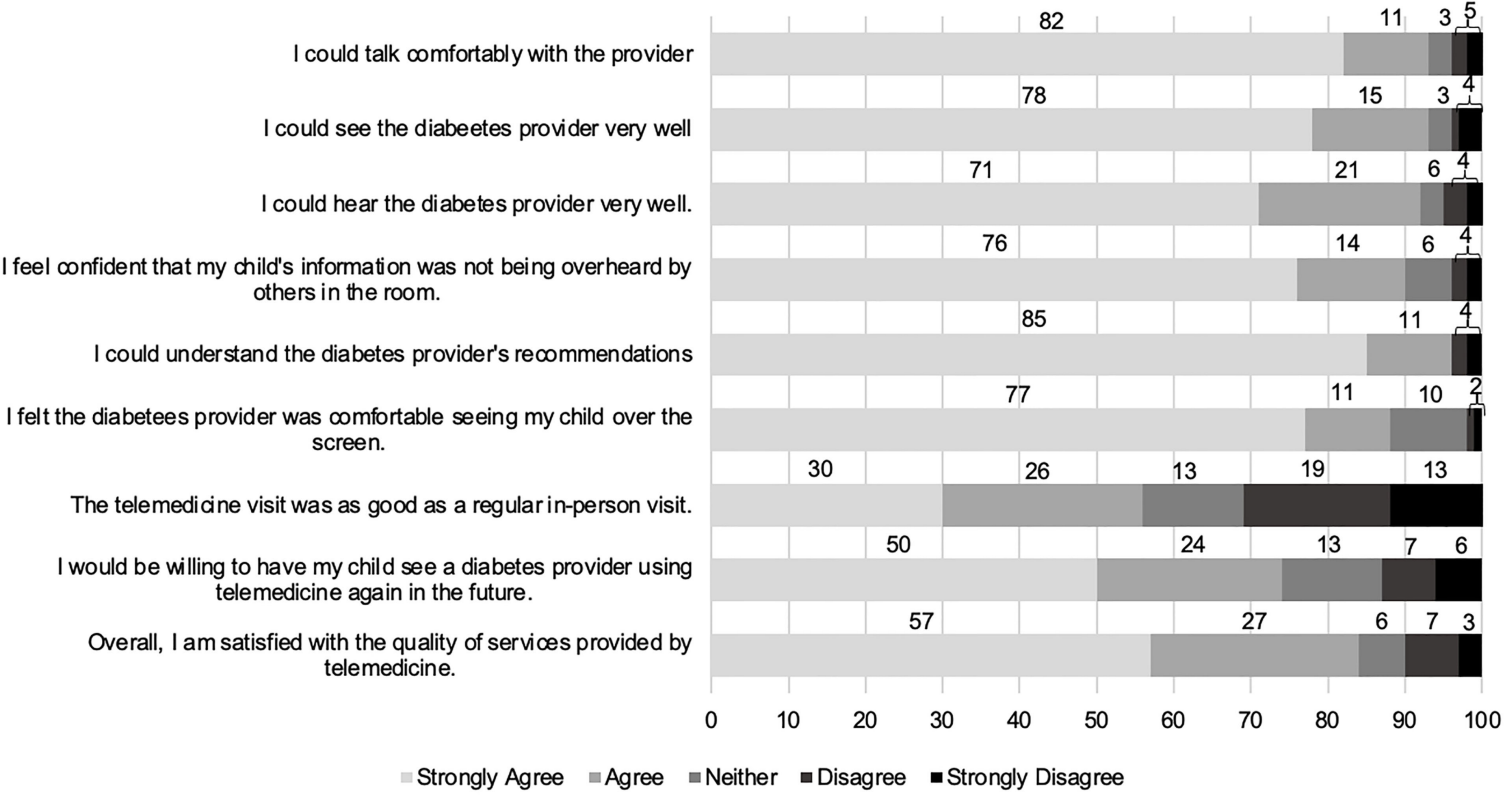
Parent Satisfaction Survey with diabetes telemedicine services: Percent of parents reporting Likert scale responses.

Parents were extremely (58%) or somewhat comfortable (25%) with virtual visits, with only 7% being uncomfortable. Parent satisfaction with telemedicine services is summarized in **Table 2** and **Figure 1**. Over 90% agreed that different aspects of the visit functioned well, though only 56% agreed the telemedicine appointment was as good as an in-person visit. Parents largely agreed (74%) that they would be willing to use telemedicine again for a diabetes appointment in the future. There were no differences in telemedicine satisfaction by age, those with or without a hemoglobin A1c in target range, pump use, or insurance status. For two questions, parents who reported having previously used telemedicine services were more likely to have a higher mean score compared to those with no prior telemedicine experience: “The telemedicine appointment was as good as a regular in-person visit” (4.11 ± 1.23 vs 3.32 ± 1.40, p=0.02) and, “I would be willing to have my child see a diabetes provider using telemedicine again in the future” (4.50 ± 1.15 vs 3.99 ± 1.20, p=0.03). Prior experience with telemedicine did not affect mean responses on other questions. Importantly, satisfaction did not differ between the two time points the survey was distributed.

**Table 2:** Parent-reported satisfaction with diabetes telemedicine visit.

Item	Mean ± *SD*
I could talk comfortably with the diabetes provider	4.68 ± 0.83
I could see the diabetes provider very well.	4.63 ± 0.86
I could hear the diabetes provider very well.	4.54 ± 0.90
I feel confident that my child’s information was not being overheard by others in the room.	4.58 ± 0.88
I could understand the diabetes provider’s recommendations	4.78 ± 0.66
I felt the diabetes provider was comfortable seeing my child over the screen.	4.61 ± 0.81
The telemedicine visit was as good as a regular in-person visit.	3.41 ± 1.41
I would be willing to have my child see a diabetes providers using telemedicine again in the future.	4.05 ± 1.2
Overall, I am satisfied with the quality of services provided by telemedicine	4.29 ± 1.03

Mean scores are out of a 5-point Likert scale from strongly agree to strongly disagree.

Parents identified obtaining lab tests such as a hemoglobin A1c (n=156, 98%), reviewing device downloads with the provider (n=149, 94%), provider conducting an exam (n=137, 86%), evaluating growth (n=131, 82%), checking injection sites (n=129, 81%), and testing the urine (n=126, 79%) during in-person visits as at least moderately important (**Table 3**). Other important factors of in-person visits cited by parents in an optional open-ended response emphasized clearer communication between the provider and family during an in-person encounter and having access to other members of the diabetes care team, including a dietician or nurse educator.

**Table 3:** Parent-rated importance of different aspects of in-person diabetes visits.

Item	Not Important	Slightly Important	Moderately Important	Very Important	Extremely Important
Checking height and weight (see how much your child grows)	11 (7)	17 (11)	52 (33)	32 (20)	47 (29)
Checking injection pump sites	9 (6)	20 (13)	43 (27)	34 (21)	52 (33)
Provider examining your child	3 (2)	19 (12)	38 (24)	42 (26)	57 (36)
Reviewing blood sugars/logs/downloads together	3 (2)	7 (4)	19 (12)	41 (25)	89 (56)
Getting lab tests (e.g. Hemoglobin A1c)	0 (0)	3 (2)	18 (11)	36 (23)	102 (64)
Getting a urine test	9 (6)	24 (15)	40 (25)	29 (18)	57 (36)

Data are reported as n(%). Small amounts of missing data may lead to total not equaling 159.

## Discussion

In our sample, parents indicated a high degree of overall satisfaction with newly instituted telemedicine services to facilitate routine pediatric diabetes care during the COVID-19 pandemic. Satisfaction was high across different functional aspects to the visit and perceived facility of the diabetes provider with telemedicine, indicating an overwhelmingly positive experience. Importantly, these findings were maintained across two survey distribution time points targeting different phases of the pandemic, indicating early and ongoing comfort with telemedicine. These findings are in line with reports from other countries which found a high degree of satisfaction with telemedicine in lieu of in-person diabetes visits during the pandemic in pediatric and adult populations (10–13). Approximately 70% of the parents in our sample would be willing to engage in future virtual visits, suggesting a role for continued telemedicine even after resolution of the pandemic. However, for successful integration of telemedicine into the current model of diabetes care, different factors will need to be considered moving forward.

Though parent satisfaction was high, over one-third of parents felt that telemedicine was not as good as an in-person visit, likely reflecting beliefs that certain aspects of in-person visits are essential. Though some of these may translate reasonably well to a virtual appointment depending upon the platform used (e.g. ability to use screen-sharing to review device downloads synchronously with families), others cannot be easily replicated, such as the physical exam. With virtual visits, patients may lose access to point-of-care testing and ancillary services (nutrition, social work, diabetes education), which may place an added burden on families to visit a laboratory or adversely impact timely care. A possible model to address these concerns could be a hybrid virtual and in-person diabetes clinic structure, where 1-2 annual visits are designated for telemedicine. Successful implementation of this model of care would require that certain criteria are met regarding clinic attendance, availability of in-person services on a reduced schedule, and the ability for families to upload devices remotely.

Second, diabetes centers must consider how various potential barriers influence equity in their approach to telemedicine, including access to technology, digital fluency, and availability of translation services. Telemedicine requires an internet-enabled device and broadband internet, both of which are less prevalent among populations already at risk for health disparities (14). In a prior study, pediatric diabetes appointment attendance during the pandemic was indeed lower among Medicaid patients and non-English speaking patients, suggesting a relationship with socioeconomic status (15). Though we found no difference in satisfaction by participant background characteristics, only three individuals who responded had no access to internet; their satisfaction scores were notably lower compared with the remaining sample. To accommodate those patients at our center with limited internet service, we ultimately offered targeted in-person visits in place of telemedicine to continue routine diabetes care. In the future, centers may endeavor to expand access to telemedicine by partnering with local primary care offices, schools, or other organizations seeking to address health disparities.

Lastly, additional logistical concerns have limited widespread use of telemedicine in the past, including legal restrictions, reimbursement concerns, and acceptance by both providers and patients (16). The CMS waivers promoted increased flexibility to engage in telemedicine, removing geographical barriers and restrictions pertaining to delivery setting, and allowed for states to provide Medicaid coverage for virtual care. Continued engagement in telemedicine will rely not only on continued regulatory changes, but also on the acceptance by both health care providers and patients. Indeed, we found that parents with prior experience with telemedicine tended to have higher satisfaction, suggesting that either personal preferences or repeated exposure may play a key role in promoting favorable experiences with telemedicine. In future research, provider perspectives need to be better elucidated and uniform strategies to measure utilization and satisfaction may help with comparisons across studies (17). Our study is strengthened by measuring satisfaction using an existing, validated scale.

Limitations of this study include the size and characteristics of the included sample. The invitation to complete the survey was distributed through our EHR, which does not allow us to track the number of messages sent or opened. Therefore, we could not identify the number of patients who received the survey and cannot accurately report a response rate. In addition, the surveyed sample may reflect a group with markers of higher socioeconomic status given the large proportion using diabetes devices and home sociodemographic factors. Given the concerns about access to telemedicine among groups at risk for disparities, additional research should specifically examine the experiences and satisfaction with telemedicine in a more diverse sample.

In summary, experiences during the COVID pandemic have offered pediatric diabetes centers a unique opportunity to redefine the standard of pediatric diabetes care, incorporating telehealth with more supportive infrastructure and training for providers. Future efforts should focus on addressing the various barriers to widespread telemedicine uptake, along with research to eliminate inequity in utilization of these services.

## Data Availability Statement

The raw data will be made available upon reasonable request.

## Ethics Statement

The studies involving human participants were reviewed and approved by the University of Pittsburgh Institutional Review Board. Written informed consent for participation was not required for this study in accordance with the national legislation and the institutional requirements.

## Author Contributions

CM and IL conceptualized the study. CM administered the survey and completed the analysis. CM, IL, and RM interpreted the results. CM drafted the initial manuscript. CM, IL, and RM edited the manuscript and approved it in its final version. All authors contributed to the article and approved the submitted version.

## Funding

CM is funded by the Children’s Hospital of Pittsburgh Scholar Award.

## Conflict of Interest

The authors declare that the research was conducted in the absence of any commercial or financial relationships that could be construed as a potential conflict of interest.

## Publisher’s Note

All claims expressed in this article are solely those of the authors and do not necessarily represent those of their affiliated organizations, or those of the publisher, the editors and the reviewers. Any product that may be evaluated in this article, or claim that may be made by its manufacturer, is not guaranteed or endorsed by the publisher.

## References

[1] De Guzman KR Snoswell CL Taylor ML Senanayake B Haydon HM Batch JA. A Systematic Review of Pediatric Telediabetes Service Models. Diabetes Technol Ther (2020) 22(8):623–38. doi: 10.1089/dia.2019.0489 32027176

[2] Reid MW Krishnan S Berget C Cain C Thomas JF Klingensmith GJ. Coyot1 Clinic: Home Telemedicine Increases Young Adult Engagement in Diabetes Care. Diabetes Technol Ther (2018) 20(5):370–9. doi: 10.1089/dia.2017.0450 29672162

[3] Lee J Carlson E Albanese-O’Neill A Demeterco Berggren C Corathers S Vendrame F. Adoption of Telemedicine for Type 1 Diabetes Care During the COVID-19 Pandemic. Diabetes Technol Ther (2021) 23(9):642–51. doi: 10.1089/dia.2021.0080 PMC8501471 33851873

[4] Wood CL Clements SA McFann K Slover R Thomas JF Wadwa RP. Use of Telemedicine to Improve Adherence to American Diabetes Association Standards in Pediatric Type 1 Diabetes. Diabetes Technol Ther (2016) 18(1):7–14. doi: 10.1089/dia.2015.0123 26295939

[5] Raymond JK Berget CL Driscoll KA Ketchum K Cain C Fred Thomas JF. Coyot1 Clinic: Innovative Telemedicine Care Model for Young Adults With Type 1 Diabetes. Diabetes Technol Ther (2016) 18(6):385–90. doi: 10.1089/dia.2015.0425 PMC5583551 27196443

[6] Koonin LM Hoots B Tsang CA Leroy Z Farris K Jolly BT. Trends in the Use of Telehealth During the Emergency of the COVID-19 Pandemic - United States, January-March 2020. MMWR Morb Mortal Wkly Rep (2020) 69:1595–9. doi: 10.15585/mmwr.mm6943a3 PMC7641006 33119561

[7] March CA Flint A DeArment D Gilliland A Kelly K Rizzitano E. Paediatric Diabetes Care During the COVID-19 Pandemic: Lessons Learned in Scaling Up Telemedicine Services. Endocrinol Diabetes Metab (2020) 2020:e00202. doi: 10.1002/edm2.202 PMC7744857 33349799

[8] Myers KM Valentine JM Melzer SM. Child and Adolescent Telepsychiatry: Utilization and Satisfaction. Telemed J E Health (2008) 14:131–7. doi: 10.1089/tmj.2007.0035 18361702

[9] Tavakol M Dennick R. Making Sense of Cronbach’s Alpha. Int J Med Educ (2011) 2:53–5. doi: 10.5116/ijme.4dfb.8dfd PMC4205511 28029643

[10] Odeh R Gharaibeh L Daher A Kussad S Alassaf A. Caring for a Child With Type 1 Diabetes During COVID-19 Lockdown in a Developing Country: Challenges and Parents’ Perspectives on the Use of Telemedicine. Diabetes Res Clin Pract (2020) 168:108393. doi: 10.1016/j.diabres.2020.108393 32858098 PMC7446666

[11] Scott SN Fontana FY Zuger T Laimer M Stettler C. Use and Perception of Telemedicine in People With Type 1 Diabetes During the COVID-19 Pandemic-Results of a Global Survey. Endocrinol Diabetes Metab (2021) 4:e00180. doi: 10.1002/edm2.180 33532617 PMC7831200

[12] Al-Sofiani ME Alyusuf EY Alharthi S Alguwaihes AM Al-Khalifah R Alfadda A. Rapid Implementation of a Diabetes Telemedicine Clinic During the Coronavirus Disease 2019 Outbreak: Our Protocol, Experience, and Satisfaction Reports in Saudi Arabia. J Diabetes Sci Technol (2021) 15:329–38. doi: 10.1177/1932296820947094 PMC7925440 32762362

[13] Isautier JM Copp T Ayre J Cvejic E Meyerowitz-Katz G Batcup C. People’s Experiences and Satisfaction With Telehealth During the COVID-19 Pandemic in Australia: Cross-Sectional Survey Study. J Med Internet Res (2020) 22:e24531. doi: 10.2196/24531 33156806 PMC7732356

[14] Katzow MW Steinway C Jan S. Telemedicine and Health Disparities During COVID-19. Pediatrics (2020) 146(2):e20201586. doi: 10.1542/peds.2020-1586 32747592

[15] Tilden DR Datye KA Moore DJ French B Jaser SS. The Rapid Transition to Telemedicine and its Effect on Access to Care for Patients With Type 1 Diabetes During the COVID-19 Pandemic. Diabetes Care (2021) 44(6):1447–50. doi: 10.2337/dc20-2712 PMC8247503 33849938

[16] Giani E Laffel L. Opportunities and Challenges of Telemedicine: Observations From the Wild West in Pediatric Type 1 Diabetes. Diabetes Technol Ther (2016) 18:1–3. doi: 10.1089/dia.2015.0360 26756102 PMC5248506

[17] Pooni R Pageler NM Sandborg C Lee T. Pediatric Subspecialty Telemedicine Use From the Patient and Provider Perspective. Pediatr Res (2021) 1–6. doi: 10.1038/s41390-021-01443-4 PMC7984505 33753896

