# Supplementary material for: How Do Virtual Visits Compare? Parent Satisfaction With Pediatric Diabetes Telehealth During the COVID-19 Pandemic
Source: Front Clin Diabetes Healthc. 2022 Jan 5;2:794493. doi: 10.3389/fcdhc.2021.794493 (PMC10012131; doi:10.3389/fcdhc.2021.794493)
Supplement: Supplementary file 1 [file Table_1.docx]

Telemedicine for Children with Diabetes during COVID-19.

DIABETES BACKGROUND

These questions are asking about your child’s diabetes.

1. How old is your child with diabetes? (drop down with ages 0– 24 years)
2. How long has your child had diabetes?
   1. Less than 1 year
   2. Between 1 and 2 years
   3. Between 2 and 5 years
   4. More than 5 years
3. What is your relationship to the child with diabetes?
   1. Mother
   2. Father
   3. Other legal guardian
4. What form of treatment does your child use for their diabetes? (check all that apply)
   1. Insulin injections using a syringe/vial or pen
   2. Insulin pump
   3. Oral medications
   4. Diet and exercise
5. Does your child use a continuous glucose monitor (e.g. Dexcom G5 or G6, Medtronic Guardian, Freestyle Libre)?
   1. Yes
   2. No

🡪 IF YES: What kind of continuous glucose monitor does your child use?

- Dexcom G5 or G6
- Medtronic Guardian
- Freestyle Libre
- Other:

1. (If insulin pump is selected for question 4) Does your child use a sensor-integrated pump (e.g. Tandem Basal IQ, Tandem Control IQ, or Medtronic 670G)?
   1. Yes
   2. No

🡪 IF YES: What kind of sensor-integrated pump does your child use?

- Tandem T Slim Basal IQ
- Tandem T Slim Control IQ
- Medtronic MiniMed 670G

1. What was your’ child’s most recent hemoglobin A1c at his/her clinic visit?
   1. <7%
   2. 7.1-8%
   3. 8.1-9%
   4. 9.1-10%
   5. >10%
   6. I am not sure

TELEMEDICINE

These questions are asking about your experience with telemedicine during the COVID-19 pandemic for your child’s diabetes care. *Telemedicine* means a health appointment for your child’s diabetes with their medical provider that is done by video or by phone.

1. PRIOR TO COVID-19, have you ever used telemedicine for your child’s diabetes or any other type of health care appointment?
   1. Yes
   2. No
2. Did your child have any telemedicine diabetes appointments during COVID-19?
   1. Yes
   2. No 🡪 end of block
3. Who made the appointment?
   1. Parent/legal guardian
   2. Child with diabetes (if over 18 years of age)
   3. Diabetes center
4. What kind of device did you use to access the telemedicine visit?
   1. Desktop or laptop computer
   2. Tablet
   3. Smart phone
   4. Regular phone – no video option
5. What kind of internet access did you have for the telemedicine visit?
   1. No internet access / Cell service only
   2. Dial-up internet
   3. High-speed / broad band internet
   4. I’m not sure
6. Did you have access to your child’s medical chart through the CHP/UPMC patient portal?
   1. Yes
   2. No
   3. I’m not sure
7. How comfortable were you with a telemedicine visit instead of an in-person visit?
   1. Extremely uncomfortable
   2. Uncomfortable
   3. Neutral
   4. Comfortable
   5. Extremely comfortable
8. Please answer the following questions about how satisfied you were with your child’s telemedicine appointment on the scale from strongly disagree to strongly agree.

|  | Strongly disagree | Disagree | Neutral | Agree | Strongly Agree |
| --- | --- | --- | --- | --- | --- |
| I could talk comfortably with the specialist. |  |  |  |  |  |
| I could see the specialist very well. |  |  |  |  |  |
| I could hear the specialist very well. |  |  |  |  |  |
| I feel confident that my child’s information was not being overheard by others in the room. |  |  |  |  |  |
| I could understand the specialist’s recommendations. |  |  |  |  |  |
| I felt the specialist was comfortable with seeing my child over the screen. |  |  |  |  |  |
| The telemedicine visit was as good as a regular in-person visit. |  |  |  |  |  |
| I would be willing to have my child see a specialist using telemedicine again in the future. |  |  |  |  |  |
| Overall, I am satisfied with the quality of services provided by telemedicine. |  |  |  |  |  |

1. Telemedicine diabetes visits are different in some ways from in-person visits. Please rate how important you feel these aspects of a diabetes visit are to your child’s diabetes care.

|  | Not all important | Somewhat important | Moderately important | Very important | Extremely important |
| --- | --- | --- | --- | --- | --- |
| Checking height and weight (see how much your child grew) |  |  |  |  |  |
| Checking injection/pump/CGM sites |  |  |  |  |  |
| Provider examining your child |  |  |  |  |  |
| Reviewing blood sugars/logs/downloads together |  |  |  |  |  |
| Getting lab tests (example: hemoglobin A1c) |  |  |  |  |  |
| Getting a urine test |  |  |  |  |  |
| Other: |  |  |  |  |  |

DEMOGRAPHICS

1. What state do you live in? (drop-down)
2. Has your child been diagnosed with COVID-19?
3. Yes, confirmed by test
4. They had symptoms of COVID-19 but were not tested
5. No

If yes or they had symptoms:

1. Was your child admitted to the hospital overnight with COVID-19?
   1. Yes
   2. No
   3. Prefer not to say
2. Have any of the following things been stressful for you during the COVID-19 pandemic? (check all that apply)
   1. Working from home while managing my child’s diabetes
   2. Working from home while helping my child with their schoolwork
   3. Having more than 1 child in distance learning
   4. Household member with a COVID-19 exposure or diagnosis
   5. Household member lost their job due to COVID-19
   6. Other:____________
   7. None of the above
3. How many people live in your home? (open-ended)
4. What is the highest level of parental education in the home?
5. Some high school
6. High school diploma / GED
7. Some college
8. College degree (Associate, Bachelor’s)
9. Graduate degree
10. Prefer not to say

1. What is the combined yearly family income?
2. Less than $5000
3. $5000 - $19,999
4. $20,000 - $49,999
5. $50,000 - $99,999
6. $100,000 - $149,999
7. Greater than $150,000
8. Prefer not to say
9. What kind of medical insurance does your child have?
10. Private
11. Public
12. Private with secondary public insurance
13. No insurance
14. Prefer not to say
15. What is your child’s race/ethnicity?
16. American Indian or Alaska Native
17. Asian
18. Black or African American
19. Caucasian
20. Hispanic
21. Two or more races
22. Prefer not to say
23. What is your child’s gender?
24. Male
25. Female
26. Trans male
27. Trans female
28. Non-binary/Gender-fluid
29. Prefer not to say
